# Wine Yeast Engineering for Improved Polysaccharide Depolymerization and Reduced Urea Accumulation

**DOI:** 10.3390/foods15214003

**Published:** 2026-11-08

**Authors:** Egor A. Vasyagin, Valery N. Urakov, Maksim Yu. Shalamitskiy, Sofia N. Cherviak, Karina A. Semenova, Natalia Yu. Lutkova, Alexey V. Beletsky, Andrey L. Rakitin, Eugenia S. Mardanova, Vitaly V. Kushnirov, Nikolai V. Ravin, Andrey V. Mardanov

**Affiliations:** 1 Institute of Bioengineering, Research Center of Biotechnology of the Russian Academy of Sciences, 119071 Moscow, Russia; egor.vasyagin@gmail.com (E.A.V.); mortu@yandex.ru (A.V.B.); rakitin@biengi.ac.ru (A.L.R.); mardanovaes@mail.ru (E.S.M.); nravin@mail.ru (N.V.R.); 2 Bach Institute of Biochemistry, Research Center of Biotechnology of the Russian Academy of Sciences, 119071 Moscow, Russia; valery.urakov@gmail.com (V.N.U.); vvkushnirov@googlemail.com (V.V.K.); 3 The National Research Centre “Kurchatov Institute”, 123182 Moscow, Russia; mshalamitskiy@yahoo.com (M.Y.S.); sofi4@list.ru (S.N.C.); karina.semenova.2013@mail.ru (K.A.S.); lutkova1975@mail.ru (N.Y.L.)

**Keywords:** wine strain, *Saccharomyces cerevisiae*, *PGU1*, *CAR1*, CRISPR/*Cas9*, polygalacturonase, urea

## Abstract

Engineering wine yeasts to combine efficient extracellular polysaccharide degradation with reduced urea accumulation may support improvements in winemaking. This study aimed to replace the arginase-encoding *CAR1* locus in the genome of the triploid industrial wine yeast *Saccharomyces cerevisiae* I-328 with an expression cassette for the endopolygalacturonase gene *PGU1*, placed under the control of the *PGK1* gene promoter and terminator. CRISPR/Cas9-mediated genome editing was performed using an acetamidase-selectable plasmid followed by phenotypic screening and molecular genetic verification. Whole genome sequencing confirmed the replacement of all three *CAR1* alleles by the *PGU1* expression cassettes. The modified strain I-328-Y21 showed enhanced degradation of polygalacturonic acid relative to the parental strain. Consistently, RT-qPCR analysis showed that the transcription level of the *PGU1* gene in strain I-328-Y21 was about 300 times higher than in I-328. Disruption of arginase—the enzyme that generates urea from arginine—reduced urea content in the growth medium by about ninefold. As ethyl carbamate is formed from ethanol and yeast-excreted urea, this strain could help lower ethyl carbamate in wine. Fermentation performance and other technological characteristics remained unaffected by the genome editing. The new strain, in which the arginase gene is replaced by an endopolygalacturonase expression cassette, is a promising candidate for winemaking.

## 1. Introduction

Pectin substances are a group of complex polysaccharides that are found in varying amounts in all tissues of higher plants. These polysaccharides mainly include polymer fragments of various molecular weights that contain 1→4-linked α-D-galacturonic acid residues partially esterified with methyl groups. Pectins pose a significant problem for the wine industry, as their presence complicates the filtration process during the purification and clarification of must and wines. Therefore, fruits in many wineries are macerated using special enzymes, namely pectinases, before the start of alcoholic fermentation. The key component of the enzymatic pectinolytic complex is endopolygalacturonase. This enzyme (EC 3.2.1.15, systematic name (1→4)-α-D-galacturonan-glycanohydrolase) hydrolyzes α-1,4-glycoside bonds between galacturonic acid residues. The use of pectinolytic enzymes in winemaking accelerates must maceration, increases the volume of released juice, reduces pressing time, facilitates filtration, and improves wine taste and color [1,2,3].

The pectinase activity in *S. cerevisiae* is determined by the *PGU1* gene, which encodes a secreted endopolygalacturonase (361 a.a.). This gene is found in most strains [4,5,6]. Endopolygalacturonase hydrolyzes polygalacturonic acid, which is present in the natural plant substrate of *S. cerevisiae* and is part of the protective structural barrier of plants against microbial invasion. The expression of the *PGU1* gene can be activated in the presence of pectin and its hydrolysis products. Endopolygalacturonase is necessary for pseudohyphal growth, characteristic of yeast and other types of fungi. This growth is a reaction to nutrient restriction and is regulated by MAPK signaling pathways [7]. It has been shown that the ability to cleave polygalacturonic acid varies in different strains of *S. cerevisiae*. It turned out that the corresponding activity is determined not by differences in the amino acid sequence of endopolygalacturase, but primarily by the transcription level of the *PGU1* gene. Fourteen genes encoding inhibitors of polygalacturonase activity, as well as two activators, were identified [8]. Another feature of the *PGU1* gene is its subtelomeric localization, and as is known, the expression of genes located near telomeres in *S. cerevisiae* is suppressed compared to the rest of the genome. It was also shown that when *PGU1* was moved to another site located far from the telomere, its transcription and corresponding pectinase activity increased [9].

As a rule, industrial wine strains of *S. cerevisiae* do not provide by themselves a level of pectinase activity sufficient for effective extraction of compounds contained in the skin of grape berries during maceration and the clarification of wine after ripening. Wine yeasts with enhanced polygalacturonase activity could provide additional pectinolytic activity during alcoholic fermentation, complementing commercial enzyme treatments applied during grape maceration, which may precede yeast inoculation. Thus, the use of a recombinant strain capable of overexpressing the *PGU1* gene and secreting endopolygalacturonase into the medium made it possible to significantly reduce the time required for wine filtration [5]. In addition, the USC-1 recombinant strain of *S. cerevisiae* with increased endopolygalacturonase activity, obtained by transformation with a plasmid carrying the *PGU1* gene, was described. The use of this strain improved the processes of clarification and filtration of wine without increasing the methanol content in these wines or changing their flavor [10]. Another study reported an industrial strain of *S. cerevisiae* containing a hybrid *PGU1* gene under the control of the constitutive promoter of the 3-phosphoglycerate kinase *PGK1* gene, integrated into the intergenic chromosomal region upstream of the *ILV2* gene. The resulting strain had an increased ability to hydrolyze polygalacturonic acid. When comparing the recombinant and the original strains under microvinification conditions, it was found that changes in the content of pectin polysaccharides did not affected the taste or aroma of the wine. It is believed that the main advantage of using such a modified strain is an increased yield of must/wine, as well as a positive effect on some variables relating to appearance [1].

Another problem encountered in winemaking is the accumulation of ethyl carbamate (EC), a group 2A carcinogen, in wine. Consequently, many countries strictly regulate the maximum allowable content of this substance in food products [11,12,13,14]. EC is formed in wine must as a result of the interaction of ethanol with urea excreted from yeast cells. Therefore, the most effective way to reduce EC content is to decrease the urea concentration. This can be achieved by reducing arginase activity through deletion of the CAR1 gene encoding arginase, which forms urea and L-ornithine via the breakdown of arginine. [11,15,16,17,18,19,20,21,22,23].

Genome editing is particularly relevant for the genetic engineering of industrial wine yeasts, since modifying them can be challenging because they are often polyploid and the desired change must be introduced into every copy of the target locus. CRISPR/Cas9 supports targeted genome modification through site-specific DNA cleavage and donor-directed repair, while selectable markers and phenotype-based screening can help identify clones carrying modifications across multiple alleles [24,25].

Previous engineering efforts have addressed enhanced pectinolytic activity and reduced ethyl carbamate formation separately. Here, we used CRISPR/Cas9 engineering to develop a dual-purpose derivative of the wine yeast strain *S. cerevisiae* I-328 by replacing the *CAR1* gene with a constitutively overexpressed *PGU1* cassette. This single-locus modification was designed to enhance endopolygalacturonase activity while reducing urea formation, which, in turn, may reduce ethyl carbamate accumulation. The triploid wine strain *S. cerevisiae* I-328 from the Magarach Collection of Microorganisms for Winemaking was used as the recipient for genome editing. General information about this strain and its genome sequence have been described previously [26,27]. The resulting strain was characterized using microbiological and molecular genetic approaches, and its fermentation performance was evaluated under experimental winemaking conditions.

## 2. Materials and Methods

### 2.1. Strains, Media and Cultivation Conditions

The *S. cerevisiae* strain I-328 was used as the recipient for genome editing. *Escherichia coli* DH10B strain, used for plasmid propagation, was cultivated in LB medium at 37 °C. Yeasts were cultivated aerobically with shaking at 30 °C in YPD medium containing 10 g/L yeast extract, 20 g/L peptone, and 20 g/L glucose, or in synthetic medium containing 1.7 g/L yeast nitrogen base (YNB) without ammonium sulfate and 20 g/L glucose. For selection, acetamide was added at a concentration of 3 g/L as the sole nitrogen source to YNB without ammonium sulfate (SC + acet). Transformation of yeast was performed using the lithium acetate method, following the protocol described by Gietz and Woods [28]. *Kluyveromyces marxianus* U848 from the Magarach Collection of Microorganisms for Winemaking [29] was used as a positive control in the polygalacturonase assay.

### 2.2. Construction of the PGU1 Expression Cassette and a CRISPR/Cas9 Vector for Integration into the CAR1 Gene of Yeast Strain I-328

Table 1 and Table 2 list oligonucleotides and plasmids employed in this work. PCR amplification was carried out using Phusion polymerase (Thermo Fisher Scientific, Waltham, MA, USA). The resulting PCR fragments were resolved by agarose gel electrophoresis and purified with the QIAquick Gel Extraction Kit (Qiagen GmbH, Heiden, Germany).

First, the CR1 fragment (207 bp), located upstream of the *CAR1* gene, and the CR2 fragment (344 bp), located downstream of this gene, were amplified by PCR using primer pairs P1/P2 and P3/P4, respectively (Table 1). The chromosomal DNA of the I-328 strain was used as a template. The resulting PCR products were then joined by overlap-extension PCR using primers P1/P4, yielding the 586-bp CR1_2 fragment. This fragment contains an *Eco47III* endonuclease recognition site at the 3′ end, as well as endonuclease recognition sites *XmaI*, *NruI*, and *XhoI* between the CR1 and CR2 regions. The PCR fragment was cloned into the *SmaI* site of the pUC19 vector, resulting in the plasmid pUC_CR1_2.

The *PGU1* gene was amplified by PCR using primers P8/P9 and chromosomal DNA of the I-329 strain as a template. The promoter and terminator regions of the phosphoglycerate kinase gene (*PGK1*), designated *P_pgk1_* and *T_pgk1_*, respectively, were amplified from chromosomal DNA of the *S. cerevisiae* strain I-329 [26] using primer pairs P5/P7 and P10/P11, respectively. The three fragments were joined by overlap-extension PCR using primers P5/P11, yielding a 2040-bp *Ppgk_PGU1_Tpgk* expression cassette containing an *NruI* site at the 5′ end and an *XhoI* site at the 3′ end. Then, this fragment was digested with *NruI* and *XhoI* endonucleases, and cloned at the same sites into the plasmid pUC19_CR1_2. As a result, plasmid pUC19_CR1_2_P_pgk__PGU_T_pgk_ (5290 bp) containing a P_pgk__PGU_T_pgk_ cassette flanked at the 5′ end of CR1 and at the 3′ end of CR2 was obtained. To obtain a linear donor DNA fragment containing the *PGU1* cassette for genome editing, a fragment of this plasmid was amplified by PCR using the P1/P4 primer pair.

CRISPR/*Cas9* editing of strain I-328 was performed using the previously described pWS-*amdS* vector, which encodes the Cas9 nuclease and contains the AmdS selective marker, which ensures the growth of yeast transformants on minimal synthetic medium with acetamide [22,31]. To construct a *CAR1*-targeting derivative, the vector was digested with *Bsm*BI to remove the *GFP* dropout cassette. Complementary oligonucleotides P12/P13 (Table 1), containing *Bsm*BI-compatible overhangs and the 20-nt spacer ACTACTACCAAGATGGAAAC, were annealed and inserted into the digested vector. The resulting plasmid, pWS-*amdS*-CAR_G, encoded a targeting RNA (sgRNA) directing Cas9 cleavage at the *CAR1* locus.

### 2.3. Genome Editing

Genome editing of the *S. cerevisiae* I-328 strain was performed using a modified yeast CRISPR/Cas9 protocol described previously [22,31]. The pWS-*amdS*-CAR_G vector was used to direct a Cas9-mediated double-stranded break in the *CAR1* locus. Strain I-328 was co-transformed with this vector and a 2626-bp linear donor DNA fragment containing the *PGU1* expression cassette flanked by regions homologous to the sequences upstream and downstream of *CAR1*. The donor fragment was designed to mediate replacement of the *CAR1* locus through homologous recombination. Transformation was carried out as previously described [28]. Transformants were selected on solid SC + acet medium lacking ammonium sulfate and containing 3 g/L [0.3% (*w*/*v*)] acetamide as the sole nitrogen source. Primary screening of putative genome-edited clones was performed by PCR using primers P1/P9 (Table 1). Clones carrying the desired modification yielded a 2061-bp amplicon. PCR-positive clones were subsequently subjected to molecular genetics and functional assays.

### 2.4. Pectinase Activity Plate Assay

The obtained clones were screened for pectinase activity using a qualitative plate assay [8]. Aliquots (10 µL) of overnight yeast cultures were spotted onto plates containing the following medium: 6.7 g/L yeast nitrogen base (YNB), 12.5 g/L polygalacturonic acid, 6.8 g/L Na_2_HPO_4_, 10 g/L glucose, and 20 g/L agar (pH 4.0). Plates were incubated at 30 °C for 2–3 days. The grown colonies were photographed, then washed off with distilled water, and the cups were filled with 6 M HCl solution and left at room temperature for 10 min. After that, they were washed with distilled water and photographed. The assay was performed once, the clear hydrolysis zones were assessed visually, and their diameters were not measured.

### 2.5. Genome Sequencing and Assembly

A single colony of the genome-edited strain was cultivated in 5 mL of YPD medium at 30 °C for 24 h. Cells were collected by centrifugation at 4000× *g* for 5 min. Total genomic DNA was isolated using the DNeasy PowerSoil Kit (Qiagen, Hilden, Germany) in accordance with the manufacturer’s protocol. DNA libraries were prepared using the NEBNext Ultra II DNA Library Prep Kit (New England Biolabs, Ipswich, MA, USA) according to the manufacturer’s recommendations and sequences on an Illumina MiSeq platform (Illumina, San Diego, CA, USA). The sequencing run generated 2,872,500 read pairs with a read length of 2 × 300 bp. Adapter sequences were removed using Cutadapt v.4.0 [32], and low-quality bases were trimmed using Sickle v.1.33 [33]. The quality-filtered Illumina reads were assembled de novo using SPAdes v.3.15.4 in isolate mode [34]. The quality-filtered reads were also aligned to the reference genome assembly of the parental strain *S. cerevisiae* I-328 (GenBank PEJR00000000) using Bowtie 2 v.2.3.5.1 [35]. Inspection of read alignments and the assembled contigs at the *CAR1* locus was used to confirm the insertion of the *PGU1* expression cassette and the deletion of the *CAR1* gene.

### 2.6. Real-Time PCR (RT-qPCR) Analysis of PGU Expression

Total RNA was extracted from yeast cells harvested during the logarithmic growth phase using the Genta RNA Isolation Kit (GenTerra, Moscow, Russia). cDNA was synthesized using the Genta-RT Kit (GenTerra, Moscow, Russia). Quantitative PCR was performed using 1 μg of cDNA, a SYBR Green reaction mixture containing SynTaq DNA polymerase (Syntol, Moscow, Russia), and gene-specific primers on a CFX96 Real-Time PCR Detection System (Bio-Rad Laboratories, Hercules, CA, USA). The cycling conditions were as follows: initial denaturation at 95 °C for 5 min, followed by 40 cycles of 95 °C for 15 s and 60 °C for 40 s. The *ALG9* gene was used as the reference gene for normalization [36]. Primer pairs P14/P15 and P16/P17 were used to amplify *ALG9* and *PGU1*, respectively (Table 1). Relative transcript levels were calculated using the 2^−ΔΔCt^ method, with the mean ΔC_t_ of the parental strain I-328 used as the calibrator. Three biological replicates were analyzed per strain, each measured in technical triplicate.

Statistical significance was assessed using a two-tailed unpaired Student’s *t*-test applied to ΔC_t_ values from three biological replicates per strain.

### 2.7. Quantitative Polygalacturonase Activity Assay

Single colonies of the parental strain I-328 and its genome-edited derivative were inoculated into 5 mL of yeast nitrogen base (YNB) medium without ammonium sulfate and amino acids, supplemented with 5 g/L glucose. Cultures were grown with vigorous shaking at 30 °C for 18–20 h until an optical density of OD_600_ ~ 3 was reached. Cells were removed by centrifugation at 4000× *g* for 5 min, and the culture supernatants were used for the polygalacturonase assay. A reaction mixture (0.5 mL) containing 0.25 mg polygalacturonic acid in 50 mM sodium phosphate buffer (pH 7.0) or in 50 mM sodium citrate buffer (pH 4.5) was supplemented with culture supernatant containing 0.5 µg of total protein. Hydrolysis was performed at 30 °C for 30 min and terminated by heating at 90 °C for 3 min. The reaction mixture without the culture supernatant served as a negative control. D-Galacturonic acid formed in the reaction samples was measured using the D-Glucuronic/D-Galacturonic Acid Assay Kit (Megazyme, K-URONIC, Bray, Ireland) according to the manufacturer’s instructions. One unit (U) of enzyme activity was defined as the amount of enzyme releasing 1 µmol of D-galacturonic acid per minute under the assay conditions described above. Total protein in the culture supernatants was determined using a fivefold-concentrated Bradford reagent (SERVA, Heidelberg, Germany) according to the manufacturer’s instructions. Background levels of D-galacturonic acid were determined using matched sample blanks containing the same culture supernatant and substrate, but without enzymatic hydrolysis. The background value was subtracted from the corresponding reaction value before calculating the enzyme activity. The assay was performed using three independent biological replicates per strain.

### 2.8. Urea Quantification by HPLC-MS/MS

Yeast strains were cultivated in liquid YGAG medium containing 0.17% yeast nitrogen base (YNB) without ammonium sulfate and amino acids, 0.5% arginine, 0.8% monosodium glutamate, 0.05% sodium sulfate, and 2% glucose. Cultures (3 replicates for each strain) were grown at 30 °C with shaking at 200 rpm for 4 days, until an optical density of OD_600_ ~ 5 was reached. The cells (1 mL adjusted to OD_600_ = 5) were centrifuged at 2000 rpm for 3 min, and 150 µL of 0.2 M xanthohydrol solution in ethanol and 30 µL of 1.5 M hydrochloric acid were added to 400 µL of the supernatant. The mixtures were incubated for 30 min, centrifuged at 12,000 rpm for 5 min and transferred to chromatography vials maintained at 8 °C.

Urea content was analyzed using a method modified from a published procedure involving derivatization with xanthydrol [37]. The high-performance liquid chromatography–tandem mass spectrometry (HPLC–MS/MS) system comprised an Agilent 1290 liquid chromatograph (Santa Clara, Santa Clara, CA, USA) equipped with a binary pump, an autosampler, and a column compartment, coupled to an Agilent 6460C mass spectrometer (Santa Clara, Santa Clara, CA, USA). Separation was performed on an Agilent Zorbax Eclipse XDB-C18 column (50 × 2.1 mm; particle size, 3.5 µm, Santa Clara, Santa Clara, CA, USA) at 35 °C and a flow rate of 0.4 mL/min. Mobile phase A contained 0.1%, and mobile phase B contained 0.1% formic acid in methanol.

The xanthydrol derivative of urea was detected in positive-ion reaction monitoring (MRM) mode using transition 263 → 181 *m*/*z*. The drying gas temperature and flow rate were 325 °C and 9 L/min, respectively. The nebulizer pressure was 30 psi, and the capillary voltage was 3500 V.

### 2.9. Assessment of Morphological, Cultural, Physiological, and Biochemical Characteristics of Yeast Strains

Test-tube cultures were maintained in grape must at (26 ± 0.5) °C. Cell morphology and dimensions were examined microscopically in cultures at the exponential phase; cell sizes were measured and processed from digital images using the Image Scope M software v. 12.3.3. After 30 days of cultivation, surface growth and sediment architecture were inspected visually. The type of deposit formed, along with the presence of a ring and a film on the medium surface, was recorded by visual assessment.

Sporulation was tested on Gorodkova solid medium (1.1% peptone, 0.5% NaCl, 0.25% glucose, 2% agar–agar, pH 7.3). An actively growing culture was inoculated onto the surface of Petri dishes; plates were incubated at (26 ± 0.5) °C and examined microscopically every third day for 5–6 weeks to detect spore formation.

H_2_S production was assessed on the solid nutrient medium BIGGY Agar [38].

Acetic acid production was detected by the formation of a clear halo around the inoculation zone. Strains were incubated for 72 h at (30 ± 0.5) °C on Chalk’s solid medium (0.3% yeast extract, 1% glucose, 0.3% calcium carbamate, 1.5% agar). Halo size was scored as follows: 0—0–1 mm (very weak ability); 1—1–3 mm (low ability); 2—3–4 mm (medium ability); 3—4–5 mm (high ability).

Acid and ethanol tolerance, cold and heat resistance, and sulfite resistance were inferred from the growth response of yeast cells on synthetic YPD medium (1% yeast extract, 2% peptone, 2% glucose, pH 3.4). Cold resistance was tested at 10 ± 1 °C; heat resistance at (37 ± 1) °C; acid tolerance at (26 ± 1) °C and pH 2.6; sulfite resistance at (26 ± 1) °C with a total sulfur dioxide concentration of 200 mg/L; ethanol tolerance at (26 ± 0.5) °C with 10, 12 and 14% *v*/*v* ethanol. To make the yeast response to the new conditions easier to identify, micro-inoculation was performed to give an initial cell count of 8–30 × 10^3^ per mL. Tubes were inspected daily for 5 days, and growth (present/absent) was recorded visually.

### 2.10. Evaluation of Fermentation Activity

CO_2_ output served as the measure of fermentation activity in the laboratory experiments. Pasteurized Aligoté grape must with 218 g/L sugars, 5.3 g/L titratable acids, 80 mg/L total SO_2_ and pH 3.3 was placed in flasks with fermentation locks. A two-day actively growing culture was inoculated to 2 × 10^6^ cells/mL. The flasks were held at (18 ± 1) °C and weighed every day for 30 days to track CO_2_ release. Triplicate data were averaged and expressed per 100 mL of must.

### 2.11. Experimental Winemaking Procedure

A single lot of 50 kg Müller-Thurgau grapes (208 g/L sugars; 6.7 g/L titratable acids; pH 3.2) served as the raw material. Vinification comprised destemming and crushing; SO_2_ addition to the must with 70–75 mg/L total SO_2_; introduction of a 3% *v*/*v* dilution of pure cultures of the strains under study; fermentation at (22 ± 2) °C; racking of the wine material off the yeast sediment; further fermentation until the density fell below 1.0 g/mL and the wine clarified spontaneously; settling and separation from the sediment; and sulfitation to 150 mg/L total SO_2_. The yeast inoculum was physiologically active (1.3 × 10^8^ cells/mL; 45% budding cells; 0.4% dead cells).

### 2.12. Oenological Characterization

At the end of fermentation (day 30), the wines were analyzed for ethanol content, titratable acid concentrations, residual sugars, and pH using standard methods adopted in winemaking [39]. Sugar concentrations were determined using a DIN 12791/L50 hydrometer (Schneider, Wertheim, Germany). The pH and titratable acidity were determined using an I-160 universal ion meter (TD Avtomatika, Minsk, Belarus) according to standard methods [39]. Ethanol content was determined by measuring the density of the distillate by hydrometry. All measurements were performed in triplicate.

Sensory evaluation of the wines was performed using a 100-point scoring system.

## 3. Results

### 3.1. CRISPR/Cas9-Mediated Replacement of CAR1 with a PGU1 Expression Cassette

**Figure 1:**
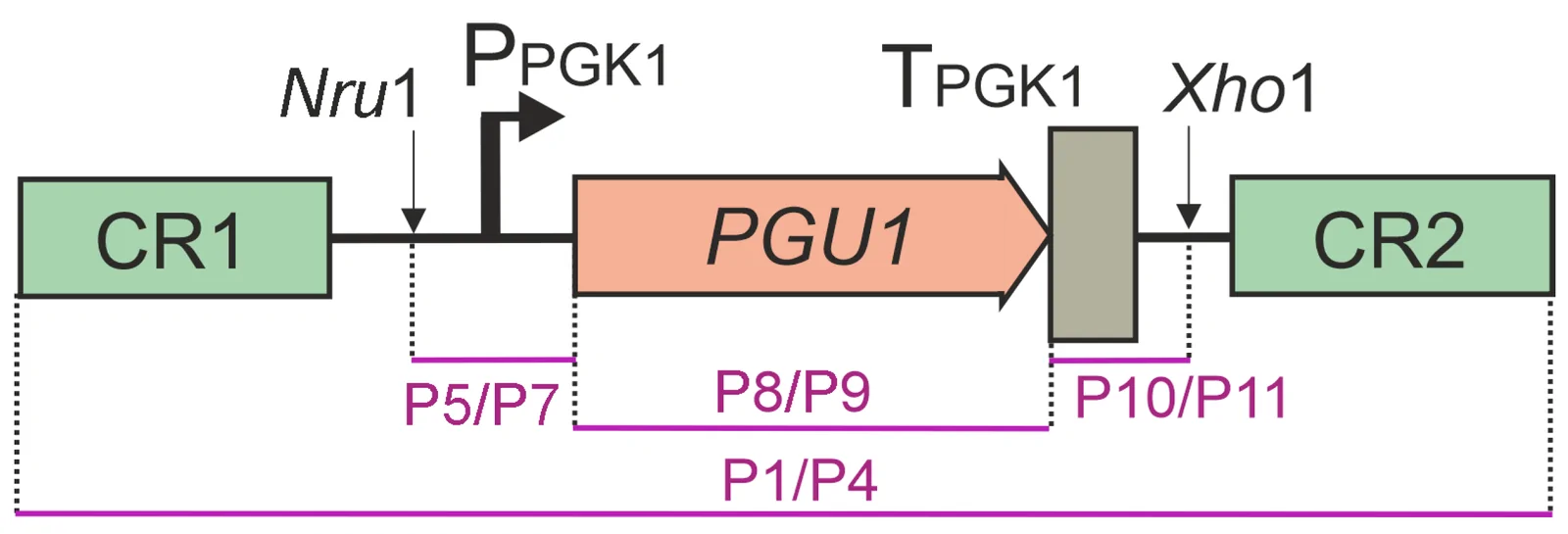
The scheme of the *PGU1* expression cassette used for genome editing. The region of integration into the genome of strain I-328 is shown. *PGU1* is the ORF of the *PGU1* gene; P_pgk1_ and T_pgk1_ are the promoter and terminator regions of the *PGK1* gene; CR1 and CR2 are fragments flanking the *CAR1* gene of the *S. cerevisiae* strain I-328.

To combine reduced urea formation with increased endopolygalacturonase expression, we replaced the arginase-encoding *CAR1* gene with the *PGU1* expression cassette. A linear donor DNA fragment containing this cassette flanked by sequences homologous to the regions upstream and downstream of *CAR1* was designed to mediate replacement through homologous recombination (Figure 1).

The wine yeast strain I-328 was co-transformed with this donor fragment and the pWS-amdS-CAR_G plasmid, which encodes *Cas9* and a *CAR1* -targeting sgRNA (see Section 2). Transformants were selected on solid SC + acet medium containing 0.3% (*w*/*v*) acetamide as the sole nitrogen source instead of ammonium sulfate.

Candidate edited clones were selected using a qualitative growth assay on a medium containing arginine as the sole nitrogen source. Loss of *CAR1* function prevents growth under these conditions [22,40]. Several transformants showed strong growth inhibition on this medium. PCR analysis confirmed the replacement of *CAR1* with the *PGU1* expression cassette, and eight clones were selected for further analysis. Selected clones were subsequently passaged several times on non-selective medium to lose the editing plasmid pWS-amdS-CAR_G.

Following the loss of the pWS-amdS-CAR G plasmid, eight selected clones were tested for pectinase activity using a qualitative plate-based assay, with *Kluyveromyces marxianus* U848 as a positive control, and the parental strain I-328 as a negative control. All tested clones demonstrated pectinase activity. Clone 8 was randomly selected from the eight pectinase-positive transformants for further characterization and designated I-328-Y21 (Figure S1).

### 3.2. Genomic Validation and Functional Characterization of Strain I-328-Y21

Because the parental strain I-328 is triploid, whole-genome sequencing was used to determine whether the targeted replacement had occurred in all *CAR1* loci in I-328-Y21. The analysis confirmed that *CAR1* was replaced by the *PGU1* expression cassette in all three copies. Analysis of the I-328-Y21 genome revealed neither additional point mutations nor genomic rearrangements.

**Figure 2:**
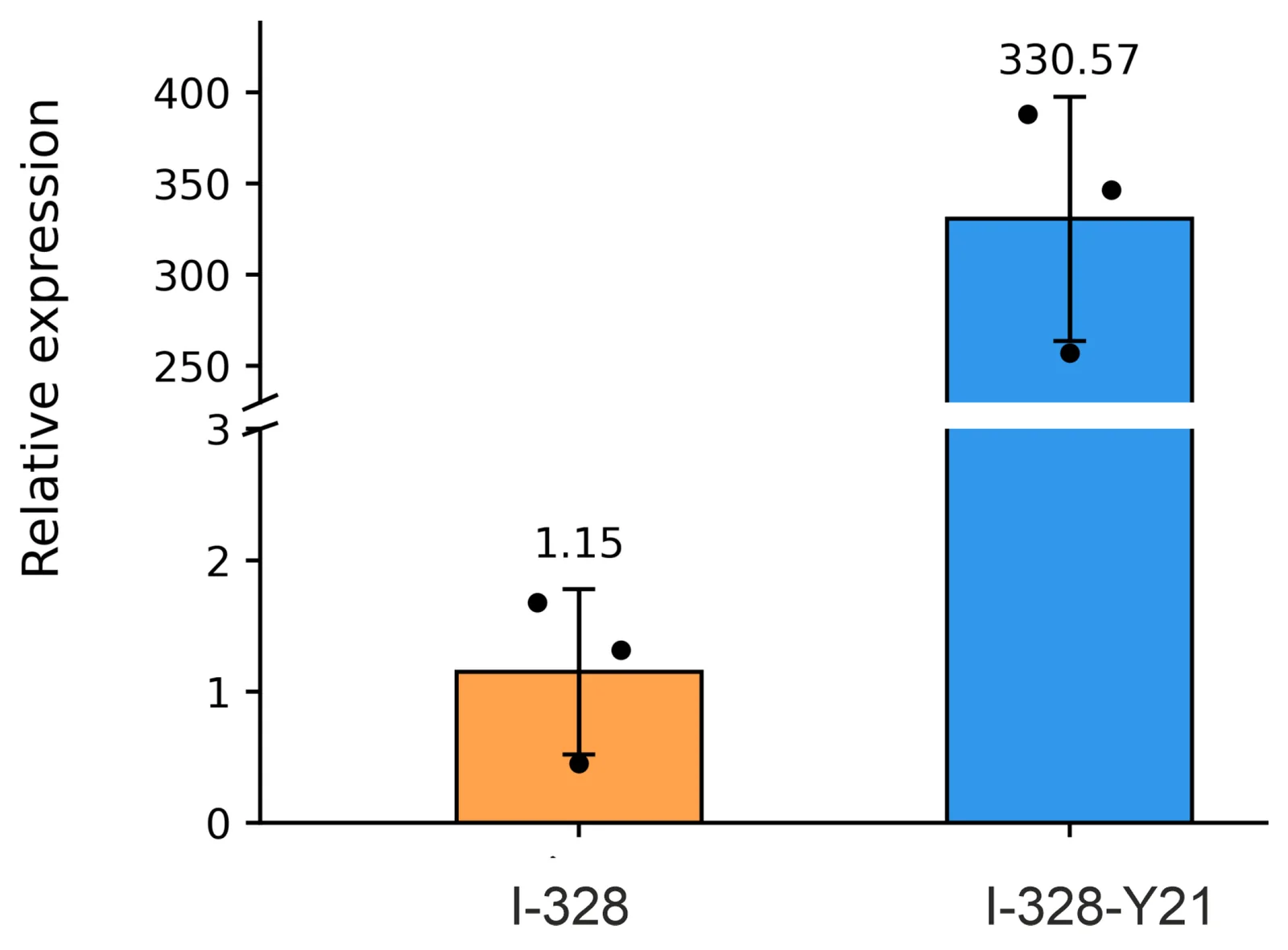
RT-qPCR analysis of relative *PGU1* transcript levels in I-328 and I-328-Y21. Data represent mean ± SD from three independent biological replicates per strain. Statistical significance (*p* = 0.00016) was assessed using a two-tailed unpaired Student’s *t*-test on ∆Ct values.

RT-qPCR analysis showed that the relative transcription level of the *PGU1* gene in strain I-328-Y21 was about 300 times higher than in I-328 (Figure 2, Table S2).

Quantitative analysis of culture supernatants showed that the specific polygalacturonase activity of strain I-328-Y21 was 4.0 ± 0.3 U/µg at pH 7.0. At pH 4.5, which is close to winemaking conditions, the activity was 2.1 ± 0.2 U/µg. No activity was detected in the parental strain I-328 under both assay conditions.

To assess the effect of *CAR1* replacement on extracellular urea accumulation, the engineered strain I-328-Y21 and the parental strain I-328 were cultivated in liquid YGAG medium at 30 °C. Urea concentrations were measured in the culture supernatants by HPLC–MS/MS (see Section 2). The extracellular urea concentration was 0.24 mg/mL for I-328-Y21, compared with 2.1 mg/mL for I-328, corresponding to an approximately 8.8-fold decrease (Table 3).

Together, the results of genome analysis, RT-qPCR, enzyme activity assays, and urea content measurements supported the integration of the *PGU1* expression cassette at the *CAR1* locus, elevated *PGU1* expression, the presence of extracellular polygalacturonase activity, and reduced urea accumulation in the culture medium of strain I-328-Y21 compared to the parental strain I-328.

### 3.3. Phenotypic, Cultural, and Biochemical Characterization of the Strains

A side-by-side evaluation of the morphological and cultural properties of I-328 and I-328-Y21 showed that the two strains share their main features (Table 4). No meaningful differences in cell shape or size were detected; only the sediment architecture varied slightly. During fermentation of must, the cells of both strains were oval to round-ovoid, as described for I-328. The sediment formed by the end of fermentation was light and weakly conglomerate, the wine clarified well, and both strains displayed weak surface growth. The parental strain sporulated, producing smooth round spores at 1–4 per ascus, whereas I-328-Y21 did not sporulate. The genetic modifications left the phenotype unchanged: the engineered strain exhibited the sensitive (S) phenotype, like the parent. Genome editing also did not affect physiological and biochemical behaviour; acetic acid synthesis was weak in both strains, with only a slight increase in hydrogen sulfide production.

### 3.4. Functional Traits of the Parental Strain and Its Engineered Variant

The fermentation potential of I-328 and I-328-Y21 was examined in pasteurized-must trials. Growth-dynamics comparison showed that CO_2_ formation by I-328-Y21 was comparable to that of the parental strain (Figure S2). Over the 15-day active fermentation period, the parent and the modified strain released 10.6 and 10.7 g/100 mL CO_2_, respectively. This result demonstrates that substituting the *PGU* cassette for *CAR1* did not affect fermentative activity.

Testing resistance to winemaking stress revealed that I-328-Y21 maintained active reproduction under different cultivation conditions for the same time intervals as the parent (Table S1), with no significant differences between the strains. Analysis of adaptation to individual abiotic factors showed the highest sensitivity of both strains to 14% *v*/*v* ethanol and the lowest sensitivity to medium acidity, high temperatures, and sulfur dioxide.

To determine the effect of the strains on product quality, wines were produced from Muller-Thurgau grapes. Data analysis confirmed that the genetic modifications performed do not affect fermentation activity. Active must fermentation was observed on the second day for both control and modified strains, and the strains did not differ significantly in fermentation rate. The obtained wines were similar in titratable acidity, residual sugar concentrations, pH and ethanol content (Table 5).

The modified strain yielded a clearer wine because colloidal compounds in the must and wine were broken down. A tasting evaluation revealed that the sample produced with the modified strain was more transparent than the control, a difference reflected in slightly higher tasting scores—81 and 80 points, respectively.

## 4. Conclusions

We developed an engineered wine yeast strain *S. cerevisiae* I-328-Y21 from the triploid parental strain I-328 by replacing all three copies of the *CAR1* locus with an overexpression cassette for the *S. cerevisiae* endopolygalacturonase gene *PGU1*. Strain I-328-Y21 retained fermentation performance similar to that of I-328 under the tested conditions, with comparable carbon dioxide release, final ethanol concentrations, and residual sugar concentrations. The engineered strain showed elevated *PGU1* expression and exhibited extracellular polygalacturonase activity, and the wine produced using this strain was clearer than the control wine.

Enhanced degradation of pectic polysaccharides could potentially reduce viscosity and colloidal haze, facilitate filtration, and improve wine clarity. Effects on phenolic extraction and color stability would require evaluation under appropriate winemaking conditions. In addition to the enhanced pectinolytic phenotype, replacement of *CAR1* was associated with an almost 90% reduction in extracellular urea accumulation after four days of cultivation in YGAG medium. Reduced accumulation of this ethyl carbamate precursor may help lower ethyl carbamate formation; however, the reduction in urea accumulation was demonstrated under laboratory cultivation conditions, and ethyl carbamate concentrations were not measured. Further validation should include direct ethyl carbamate measurements in wine. Evaluation of the technological benefits associated with the strain’s polygalacturonase activity should also include quantitative assessment of turbidity and filtration performance under winemaking conditions.

The enhanced extracellular polygalacturonase activity and reduced urea accumulation observed here are consistent with previous studies involving *PGU1* overexpression and *CAR1* deletion [1,10,16]. Direct quantitative comparison is limited by differences in cultivation conditions, enzyme assay methods, and activity normalization. Thus, the realized genome-editing strategy combined enhanced pectinolytic activity with reduced urea accumulation while maintaining similar fermentation performance under the tested conditions. This approach provides a basis for targeted improvement of wine yeasts, with further validation needed to quantify its processing benefits and effects on ethyl carbamate concentrations in wine.

## Figures and Tables

**Table 1:** Oligonucleotides used in this work.

Primer	Sequence (5′-3′)
P1	AACCGTGTAGGCAAAACTGGAC
P2	CTCGAGTCGCGACCCGGGAGACAGTATGCGTAGCTTTACCA
P3	CCCGGGTCGCGACTCGAGATCATCATCCCTTTTATCAAAATAAGCA
P4	AGCGCTAAGAAGTGGAATTTATT
P5	TATCGCGAACGCACAGATATTATAACAT
P7	AAGTAATGAATTAGCAGAAATCATTGTTTTATATTTGTTGTAAA
P8	TTTACAACAAATATAAAACAATGATTTCTGCTAATTCATTACTT
P9	CGATTTCAATTCAATTCAATTTAACAGCTTGCACCAGATC
P10	GATCTGGTGCAAGCTGTTAAATTGAATTGAATTGAAATCG
P11	ATCTCGAGAGGCATTAAAAGAGGAGCGA
P12	gactACTACTATCAAGATGGAAAC
P13	aaacGTTTCCATCTTGATAGTAGT
P14	CACGGATAGTGGCTTTGGTGAACAATTAC
P15	TATGATTATCTGGCAGCAGGAAAGAACTTGGG
P16	AGCGACATAACAATTGATATCAGAGACGGT
P17	CCTACAGAAATACCATGGCCATTGTAGCA

**Table 2:** List of plasmids used in the work.

Name	Description	Reference
pUC19	Cloning backbone used in this study	[30]
pUC19_CR1_2	pUC19 carrying the 586-bp *CAR1*-flanking assembly	This study
pUC19_CR1_2_Ppgk_PGU_Tpgk	pUC19 containing a *Ppgk_PGU_Tpgk* cassette	This study
pWS-amdS	Cas9 vector with an acetamidase gene	[31]
pWS-amdS-CAR_G	pWS-*amdS* derivative carrying the *CAR1*-targeting guide sequence	This study

**Table 3:** Urea content in culture supernatants of strains I-328-Y21 and I-328.

Strain	Urea (mg/mL) *	Relative to I-328 (%)
I-328 (control)	2.1 ± 0.2	100
I-328-Y21	0.24 ± 0.02	11.4

* Measurements were performed in three biological replicates.

**Table 4:** Comparative Morphological and Cultural Characteristics of the Strains.

Strain	Cell Morphology	Average Cell Size, µm	Sediment	Ring *	Velum *
I-328 (control)	Round-ovoid; elongated and oval ones are also found	8.33 × 5.74	Dusty	Weak	Small islands
I-328-Y21	Round-ovoid; elongated and oval ones are also found	8.53 × 6.03	Dusty	Weak	Small web

* on the surface of the wine.

**Table 5:** Chemical Profiles of the Experimental Wines.

Characteristic	I-328	I-328-Y21
Glucose, g/L	1.7 ± 0.1	1.8 ± 0.1
Fructose, g/L	1.1 ± 0.1	1.3 ± 0.1
Ethanol, % (*v*/*v*)	11.8 ± 0.05	12.0 ± 0.05
Titratable acidity, g/L	6.6 ± 0.05	6.7 ± 0.05
pH	3.2	3.2

## Data Availability

The original contributions presented in this study are included in the article/Supplementary Material. Further inquiries can be directed to the corresponding author.

## Supplementary Materials

The following supporting information can be downloaded at https://www.mdpi.com/article/10.3390/foods15214003/s1, Figure S1: Identification of transformants exhibiting pectinase activity using a qualitative plate assay; Figure S2: Fermentation activity of strains I-328 and I-328-Y21; Table S1: Resistance of strains to stressful cultivation condition; Table S2. RT-qPCR analysis of *PGU1* transcription in *S. cerevisiae* I-328 and I-328-Y21.
