# Supplementary material for: Wine Yeast Engineering for Improved Polysaccharide Depolymerization and Reduced Urea Accumulation
Source: Foods. 2026 Nov 8;15(21):4003. doi: 10.3390/foods15214003 (PMC13762605; doi:10.3390/foods15214003)
Supplement: Supplementary file 1 [file foods-15-04003-s001.zip › Figure S1.pdf]

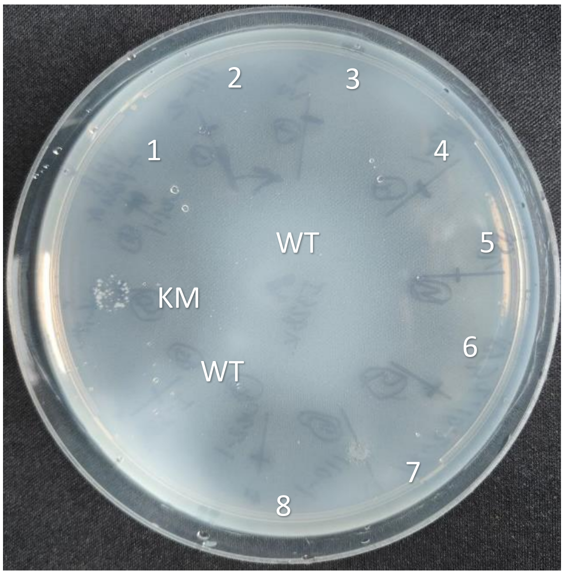

after washing with 6M HCl

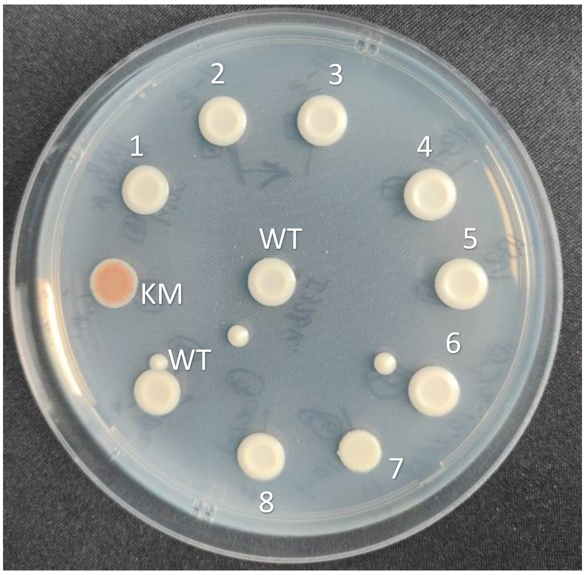

Growth on YNB with polygalacturonic acid

Figure S1. Identification of transformants exhibiting pectinase activity using a qualitative plate assay.

Pectinase activity was manifested by the appearance of a clear zone after washing with 6 M HCl. KM, *Kluyveromyces marxianus* U848 (positive control). WT, parental strain *S. cerevisiae* I-328 (negative control). Modified strains are shown by numbers (1 through 8). Strain No 8 was randomly selected and designated I-328-Y21.
