# Supplementary material for: Wine Yeast Engineering for Improved Polysaccharide Depolymerization and Reduced Urea Accumulation
Source: Foods. 2026 Nov 8;15(21):4003. doi: 10.3390/foods15214003 (PMC13762605; doi:10.3390/foods15214003)
Supplement: Supplementary file 1 [file foods-15-04003-s001.zip › Figure S2.pdf]

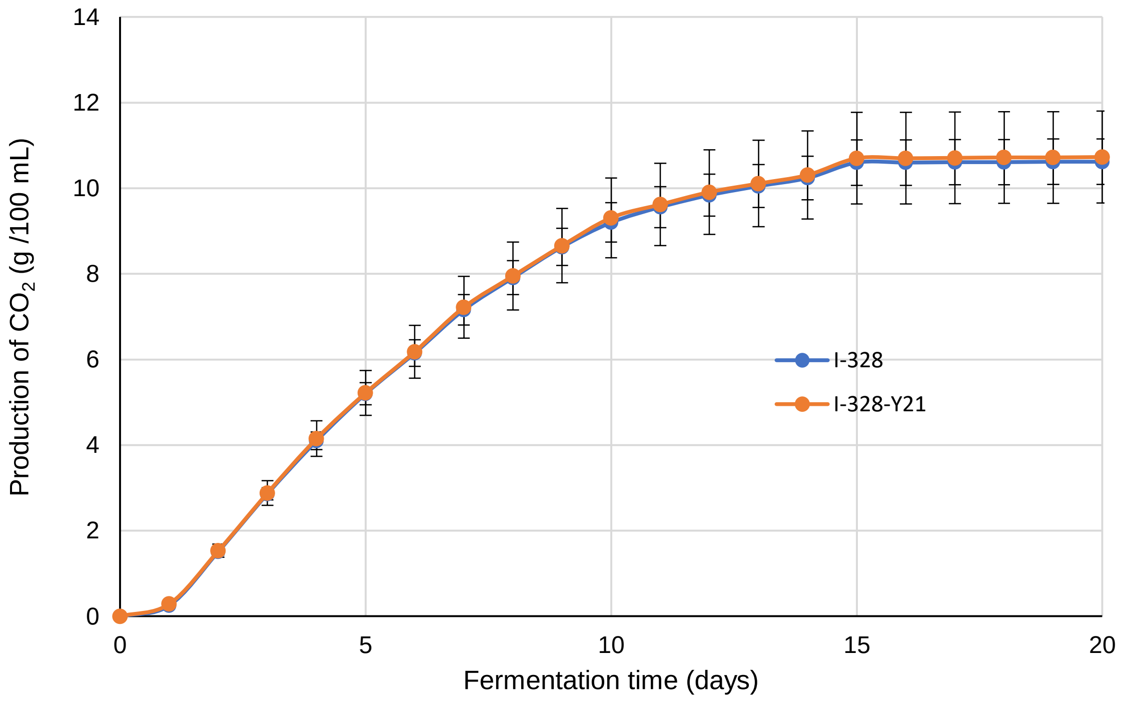

Figure S2. Fermentation activity of strains I-328 and I-328-Y21.
