# Supplementary material for: Wine Yeast Engineering for Improved Polysaccharide Depolymerization and Reduced Urea Accumulation
Source: Foods. 2026 Nov 8;15(21):4003. doi: 10.3390/foods15214003 (PMC13762605; doi:10.3390/foods15214003)
Supplement: Supplementary file 1 [file foods-15-04003-s001.zip › Table S1.pdf]

Table S1. Resistance of strains to stressful cultivation condition.

Strain	Start of growth, days after inoculation						
	Temperature 10°C	Temperature 37°C	Ethanol 10 % (v/v)	Ethanol 12 % (v/v)	Ethanol 14 % (v/v)	Concentration of sulfurous acid 200 mg/L	pH 2.6
I328-Y21	4	1	3	4	No growth	1	1
I-328	4	1	3	4	No growth	1	1
