# Supplementary material for: Wine Yeast Engineering for Improved Polysaccharide Depolymerization and Reduced Urea Accumulation
Source: Foods. 2026 Nov 8;15(21):4003. doi: 10.3390/foods15214003 (PMC13762605; doi:10.3390/foods15214003)
Supplement: Supplementary file 1 [file foods-15-04003-s001.zip › Table S2.pdf]

Table S2. RT-qPCR analysis of *PGU1* transcription in *S. cerevisiae* I-328 and I-328-Y21.

The data for three biological replicate samples are shown. The Ct values represent the means of three technical replicates for each biological sample. ΔCt was calculated as Ct(PGU1) − Ct(ALG9), with A*LG9* used as the reference gene.

Strain	Mean Ct *ALG9*	Mean Ct *PGU1*	ΔCt
**I-328**	21.9533	24.1667	2.2133
**I-328**	20.4867	24.5900	4.1033
**I-328**	21.9867	24.5500	2.5633
**I-328-Y21**	21.9133	16.2733	−5.6400
**I-328-Y21**	20.7433	15.6967	−5.0467
**I-328-Y21**	21.2033	15.7267	−5.4767
